# Percutaneous femoral de-rotational varus osteotomy for the treatment of acetabular dysplasia: surgical technique

**DOI:** 10.1051/sicotj/2023003

**Published:** 2023-02-28

**Authors:** Rami Jahmani, Ziad Ali Audat, Abdualaziz Z Alanazi, Giovanni Lovisetti

**Affiliations:** 1 Department of Orthopedic Surgery, Jordan University of Science and Technology P.O. Box 3030 Irbid 22110 Jordan; 2 Medical Student, Jordan University of Science and Technology P.O. Box 3030 Irbid 22110 Jordan; 3 Orthopaedic and Traumatology Unit, Menaggio Hospital 22017 Menaggio Como Italy

**Keywords:** Developmental dysplasia of the hip, Femoral de-rotational varus osteotomy, External fixator, Percutaneous osteotomy, Minimal invasive

## Abstract

Pediatric acetabular dysplasia is common in orthopedic practice. Femoral de-rotational varus osteotomy (FDVO) is one of the surgical options suggested for treatment. In this article, we describe a simplified surgical technique of performing FDVO percutaneously using a pediatric Limb Reconstruction System external fixator, and we discuss the advantages and disadvantages of the technique.

## Introduction

Acetabular dysplasia (AD) has been reported to be the main cause of hip arthroplasty in adults [1]. It may exist despite appropriate treatment of developmental dysplasia of the hip [2]. Two surgical options have been suggested; femoral de-rotational varus osteotomy (FDVO) and acetabular osteotomy [2, 3]. Several surgical techniques and implants have been developed to perform FDVO. Nowadays, the most frequently used implant systems are angled blade plates, lateral locking compression plates, and sliding hip screws [4–6]. Several drawbacks and complications of using internal fixations to perform FDVO have been reported, such as surgical site morbidity and skin scarring, blood loss, bone shortening due to the need for trapezoidal bone fragment removal, the inadequacy of stable fixation and need for hip spica, need for a second procedure for metal removal, infection, fracture, loss of fixation, metal failure, delayed union, and non-union [4–6]. Although external fixators are widely used in orthopedic practice, the literature is lacking detailed description of the surgical technique of FDVO using an external fixator. We use the Pediatric Limb Reconstruction System (LRS) external fixator for the treatment of AD. LRS is a monoplanar external fixator, which is used to treat leg length discrepancy and bone reconstruction. It is made of sliding clamps on a strong rail. The simplicity, versatility, and stability of the LRS make it an interesting osteosynthesis device for FDVO. In this article, we describe the surgical technique and discuss its advantages and disadvantages.

## Surgical technique

The surgery is performed with the patient under general anesthesia in the supine position. The involved limb is brought to the edge of the table.

**Figure 1:**
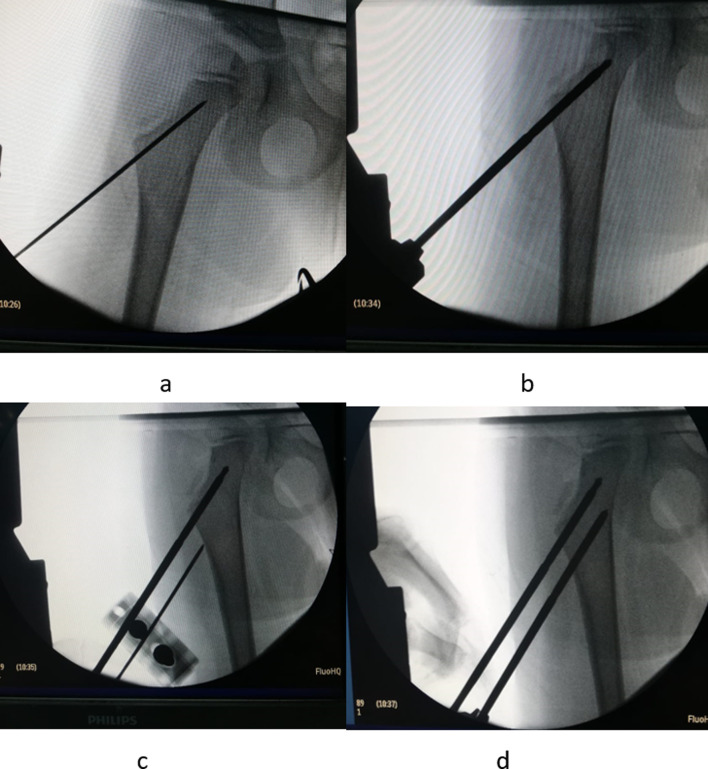
Proximal pins insertion: a K-wire is inserted in the uppermost region of the neck (a), which is replaced then by a pin (b). The second pin is inserted parallel using the external fixator clamp as a template (c, d).

*Step 1: Proximal pins insertion (**Figure 1**)*.

Under radiographic guidance, a K-wire is driven into the neck of the femur, in the uppermost region. Frog-leg position lateral radiography is then performed to confirm the correct positioning of the wire. Subsequently, the bone is drilled over the K-wire by using a cannulated drill bit, and the K-wire is then replaced by a Schanz pin of appropriate size. Then, using the clamp of the external fixator as a template, another Schanz pin parallel to the first pin is inserted in the next closest distal hole. About 4-mm Schanz pins were the most appropriate size in most cases, although the size can vary according to bone diameter.

**Figure 2:**
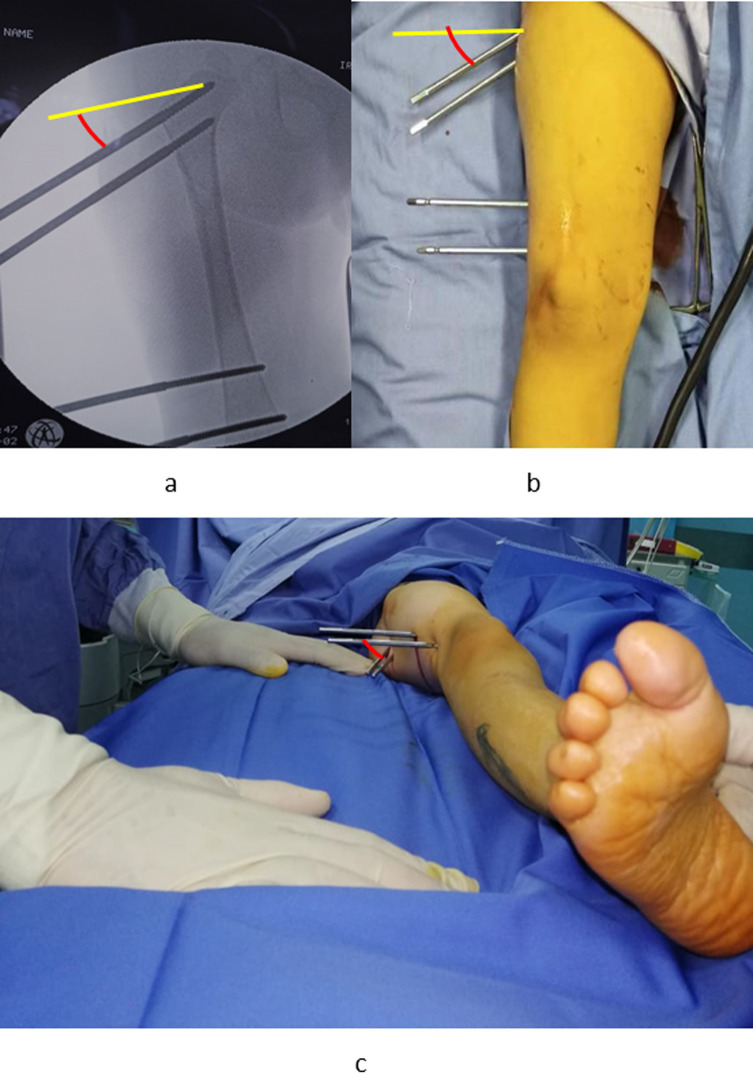
Distal pins insertion: Figure is depicting the diversion angle between proximal pins and distal pins in the frontal plane (a, b) and horizontal plane (c).

*Step 2: Distal pins insertion (**Figure 2**).*

**Figure 3:**
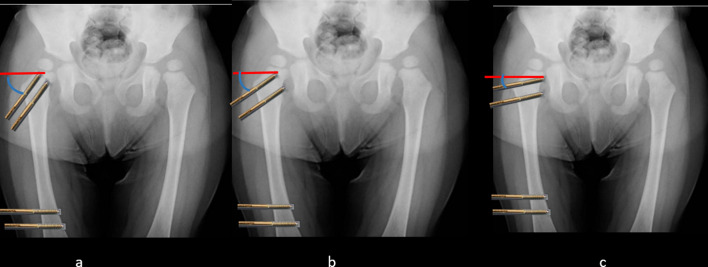
The amount of varisation: The amount of diversion angle between proximal and distal pins (Cobb angle) equals the amount of varisation. The more converted angle, the more varisation. a > b > c.

Two Schanz pins are inserted in the distal part of the bone. The diversion angle between the distal pins and proximal pins (Cobb angle) in the frontal plane equals the amount of needed varisation. If more varus is needed, more diverted pins are inserted, and vice versa (Figure 3). The same principle is applied for de-rotation in the horizontal plane. We found it easier to insert the distal pins perpendicular to the femur shaft (reference pin); while the proximal ones (neck pins) are inserted in a diverted Cobb angle. The driving of K-wires first, then replacing them with Schanz pins, decreases the risk of under/over-correction. Swiveling clamps are advised to be used when available.

**Figure 4:**
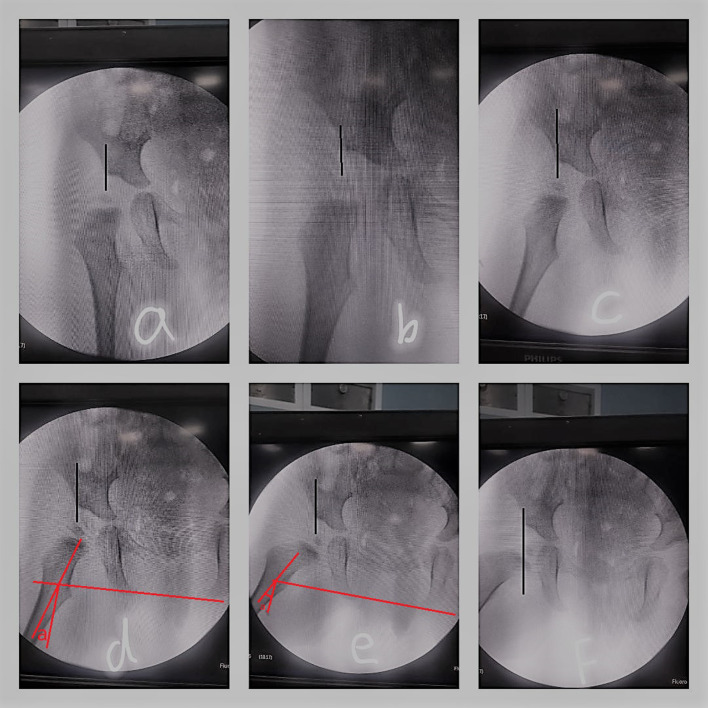
Intraoperative estimation of varisation (abduction then internal rotation of the hip to get the ossific nucleus completely covered by the acetabulum): Hip is in neutral position (a). Hip is being abducted (b, c, d). Hip is being internally rotated (e, f). Black line is Perkin’s line. “a angle” is the amount of hip abduction which was needed to make the nucleus completely covered by the acetabulum, which equals the amount of needed varus. Note that rotation was needed (e, f) to make the ossific nucleus completely inside the acetabulum.

The amount of intended varus is planned pre-operatively. We calculated the amount of varisation intra-operatively as well. The hip is abducted under radiographic guidance, and the amount of varus and de-rotation correction equals the amount of hip abduction and rotation in which the ossific nucleus becomes completely covered by the acetabulum (Figure 4).

**Figure 5:**
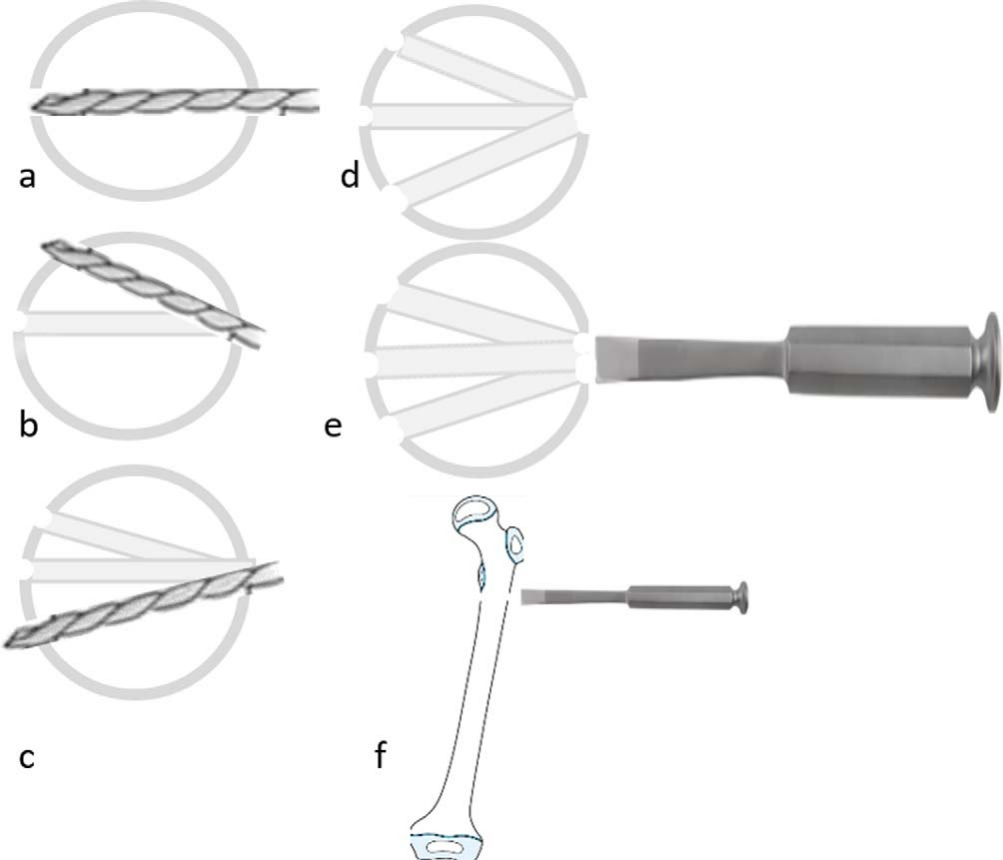
Percutaneous osteotomy: drilling the bone in different directions (a, b, c, d), then completing the osteotomy by osteotome (e, f).

*Step 3: Percutaneous bone osteotomy (**Figure 5**).*

Percutaneous osteotomy is performed just distal to the proximal pins in the sub-trochanteric area using the multiple-drill hole osteotomy technique [7]. Through a small skin incision (a stab wound), a series of multiple drill holes are made in the bone; one hole is lateral to the medial direction, and two holes are redirected in an oblique anteromedial and posteromedial direction. Following the multiple drill holes, the osteotomy is completed using an osteotome.

**Figure 6:**
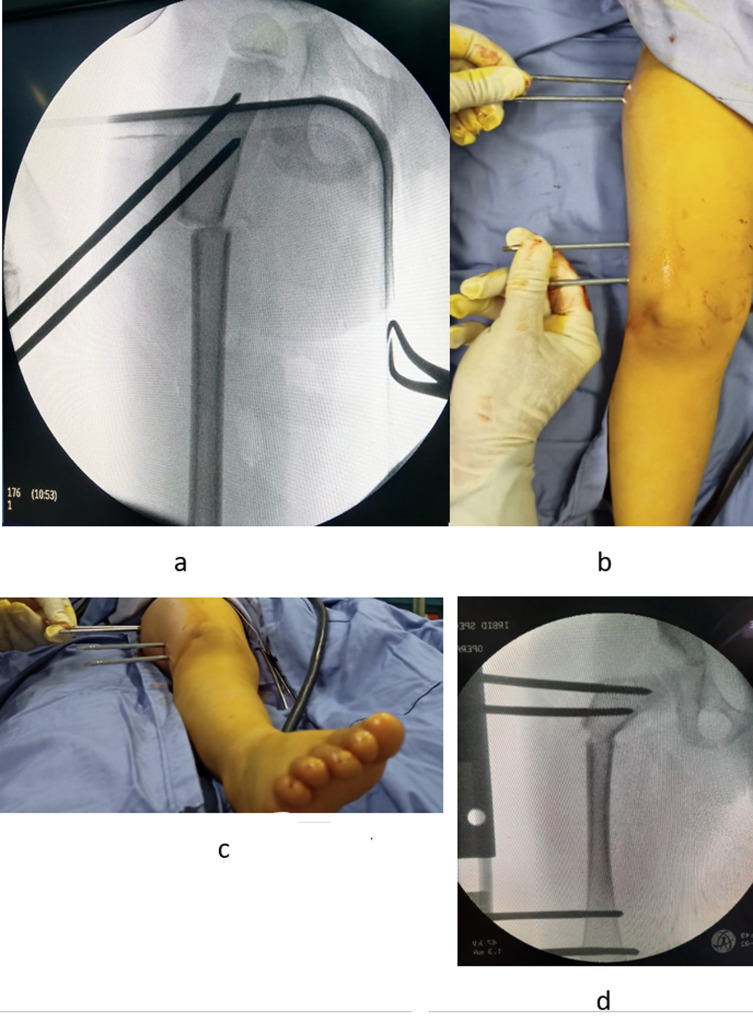
Bone shifting, de-rotation, varisation, and connection of the external fixator: Distal bone is shifted medially (a), then pins are made parallel in both frontal and horizontal planes (b, c), then external fixator is connected (d).

*Step 4: Bone shifting, de-rotation, varisation, and connection of the external fixator (**Figure 6**).*

**Figure 7:**
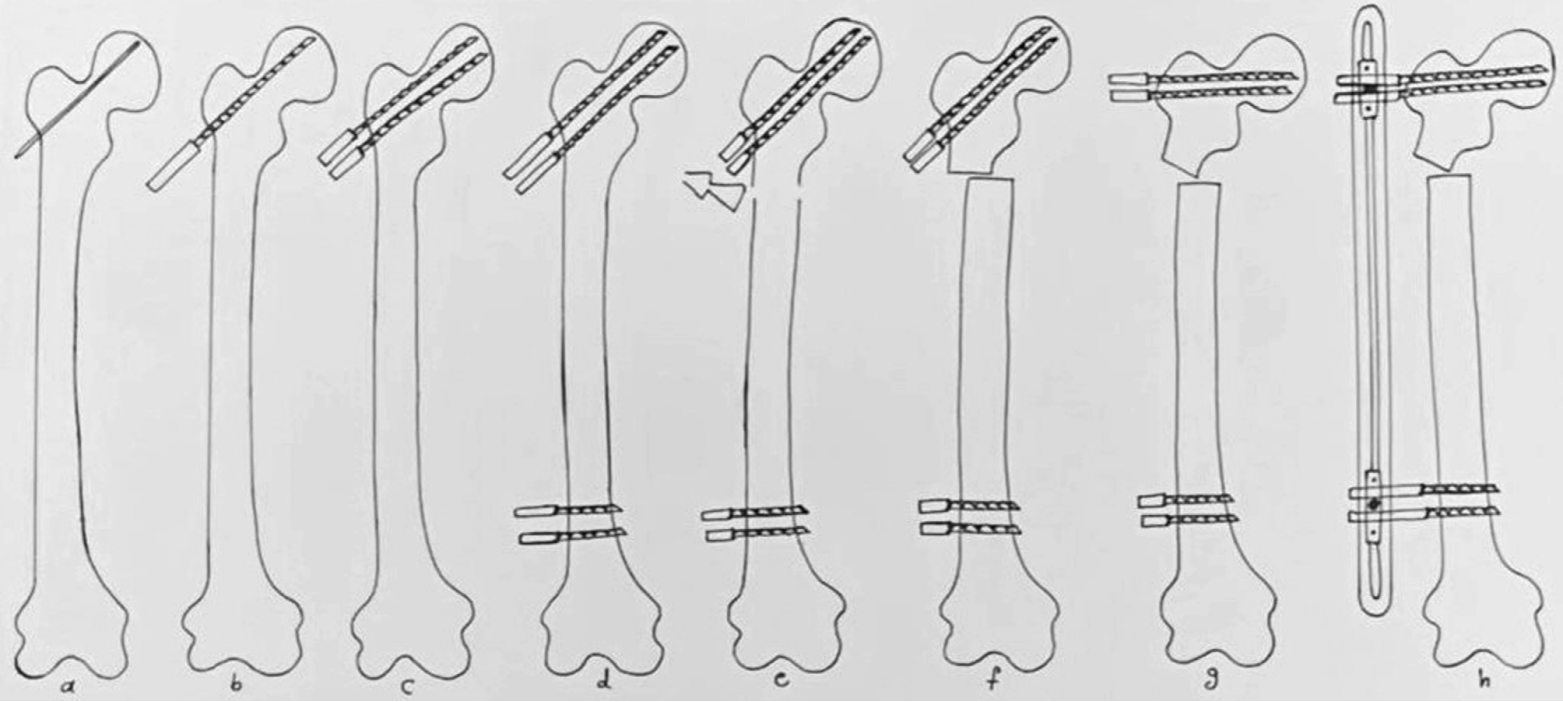
Graphical illustration summary of the technique: (a) K-wire insertion; (b) replacement of the K-wire by a pin; (c) adding one more proximal pin; (d) insertion of proximal pins; (e) performing percutaneous osteotomy; (f) bone shifting following osteotomy rule 2; (g) varisation, making the proximal pins parallel to the distal ones; (h) connecting the pins to the external fixator.

**Figure 8:**
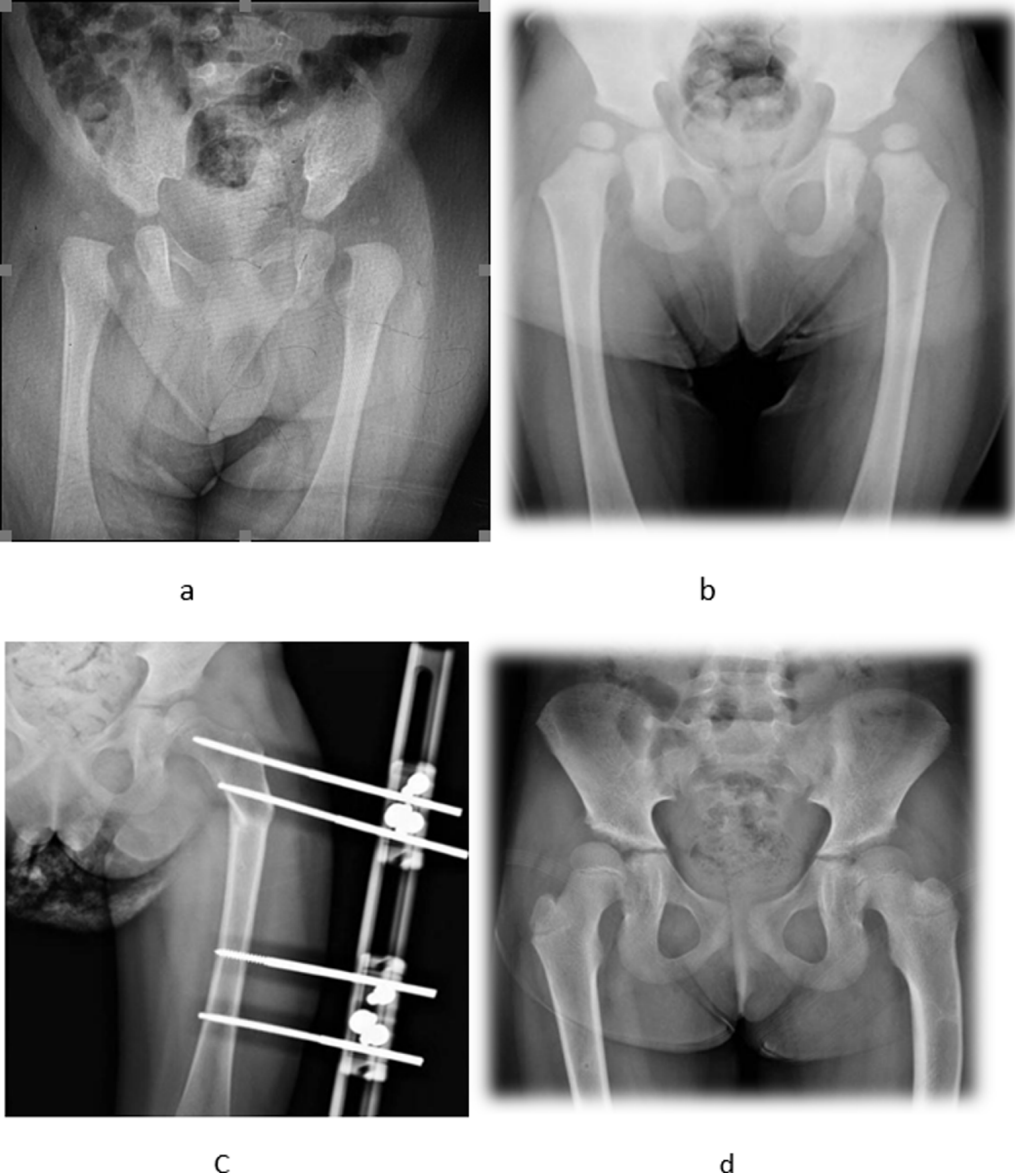
Cases example: Bilateral hip dislocation (a) was treated by Pavlic harness, ended up with left acetabular dysplasia, which persisted after a year of observation (b). FDVO was performed (c), and a good acetabular response after 2 years of follow-up (d).

Because osteotomy is performed distal to the center of rotation of angulation (CORA) of deformity (CORA is at the head-neck junction), osteotomy rule 2 is applied [7] by shifting the femoral shaft medially. Insertion of the proximal pins in the uppermost region of the neck allows performing osteotomy closer to the CORA, which decreases the amount of shifting. After medial shifting, de-rotation of the hip is performed by rotating the proximal pins internally and making them in the same plane with distal pins (Figure 6c), Subsequently, varisation is performed by moving the proximal pins and making them parallel to the distal pins. It is important to follow the subsequent order of shifting, rotation, then varisation. Performing varisation first makes bone shifting difficult because of soft tissue tensioning. Finally, the pins are connected to a monoplanar external fixator, and the clamps are locked. Different monoplanar external fixators can be used; we utilize the short (LRS) external fixator. Figure 7 demonstrates a graphical illustration summary of the technique. Figure 8 shows a case example.

*Post-operative period*

Patients were allowed to bear full weight as tolerated. Parents were taught to take care of the pins by daily washing them with clean water and soap, then covering them with clean dry gauze twice a day.

### Indication for surgery and amount of varisation

There is no conclusion in the literature so far about the optimum magnitude of varisation; moreover, the magnitude of varisation does not correlate with the final result of acetabular remodeling [8]. After performing FDVO, the remodeling ability of the proximal femur is a cause for concern. Some studies have shown how altered anatomy of the proximal femur could lead to instability and worse outcomes, requiring correction [9, 10]. However, Kasser et al. [8] reported that no patient who was less than 8 years old at the time of osteotomy failed to manifest femoral remodeling, regardless of the degree of varisation. In our practice, we decided to correct the valgus angle to the degree that results in complete coverage of the head by the acetabular roof, which was estimated intraoperatively by performing hip abduction under radiographic guidance (Figure 4).

It is important to consider the potential remodeling ability of the acetabulum when performing FDVO. Generally, it is believed that acetabulum remodeling significantly decreases after the age of 8 years [8, 11]; hence, femoral osteotomy alone is usually not recommended beyond this age. Kasser et al. [8] concluded age to be the most important predicting factor of successful results of FDVO in residual acetabular dysplasia treatment. They reported excellent and good results in most cases of acetabular dysplasia operated by FDVO before the age of 4 years; by contrast, almost half of the operations performed in patients aged >4 years resulted in fair or poor outcomes. They attributed these results to the presence of hidden subluxation of the head in children aged more than 4 years. Based on these results, we consider performing early surgery before the age of 4 years; and if hip subluxation is suspected, open reduction is considered. Indications for surgery include failure of the hip to undergo progressive development after one year of reduction, as evidenced by a decreasing acetabular index or increasing lateral center-edge angle. Another indication is any residual hip dysplasia by the age of 4 years (acetabular index above 30).

## Discussion

It has been generally agreed that FDVO is an excellent treatment for AD [2, 8]. In this article, we describe the surgical technique of performing FDVO using monoplanar external fixator, specifically the LRS fixator. This technique is simple and associated with several benefits. The percutaneous approach decreases the amount of soft tissue damage and surgical skin scarring and reduces blood loss. The closed, low-energy, multiple drill hole osteotomy technique allows performing open wedge transverse osteotomy without the need for removal of trapezoidal bone fragments and bone shortening. Low-energy osteotomy and minimal soft tissue damage are associated with a high union rate, as well. Early mobilization and elimination of hip spica use is another advantage of this technique. Patients are allowed full weight bearing immediately after surgery. The technique is simple, requires a relatively short surgical time, and eliminates the need for additional major surgery for metal removal. Another advantage is the feasibility to perform medial shifting of the distal fragment, which is not always possible to do when using internal fixation [6]. In comparison with pelvic osteotomies, the percutaneous technique of FDVO is less complicated. Pelvic osteotomies are technically demanding and several major complications have been reported.

However, there are potential limitations to this technique. The inconvenience of an external fixator and pin tract infection are the main drawbacks, for which counseling with the patient’s guardian becomes necessary. Patient education and appropriate local pin site care along with strict adherence to the principles of pin insertion decrease the incidence of pin tract infection [12].

By the end, although acetabular dysplasia is common (up to a third of patients successfully treated for DDH show residual dysplasia) [13, 14], it remains one of the most debatable conditions to manage. There is debate about the natural history, diagnosis, indication for treatment, and way of treatment [15]. With this dilemma, we prefer to follow the least invasive method of treatment.

## Conclusion

Monoplanar external fixation could be an alternative option for performing FDVO in the treatment of AD.
